# Genetic architecture of key traits for *Prunus* crop improvement: an overview of 25 years of curated genomic and breeding data

**DOI:** 10.1093/hr/uhaf142

**Published:** 2025-05-30

**Authors:** Michael Itam, Sook Jung, Ping Zheng, Taein Lee, Chun-Huai Cheng, Katheryn Buble, Dorrie Main, Ksenija Gasic

**Affiliations:** Department of Plant and Environmental Sciences, Clemson University, Clemson, SC 29634-0310, USA; Department of Horticulture, Washington State University, PO Box 646414, Pullman, WA 99164-6414, USA; Department of Horticulture, Washington State University, PO Box 646414, Pullman, WA 99164-6414, USA; Department of Horticulture, Washington State University, PO Box 646414, Pullman, WA 99164-6414, USA; Department of Horticulture, Washington State University, PO Box 646414, Pullman, WA 99164-6414, USA; Department of Horticulture, Washington State University, PO Box 646414, Pullman, WA 99164-6414, USA; Department of Horticulture, Washington State University, PO Box 646414, Pullman, WA 99164-6414, USA; Department of Plant and Environmental Sciences, Clemson University, Clemson, SC 29634-0310, USA

## Abstract

The extensive accumulation of genetic, genomic, expression, and breeding data on *Prunus* species often results in valuable information being lost or difficult to access for breeding purposes. We report a recent effort to increase curation on *Prunus* data in the Genome Database for Rosaceae (GDR, rosaceae.org) and a case study that explores 25 years of curated data (from 1998 to 2023) to uncover the genetic architecture of key traits in *Prunus* species, provide actionable insights for breeding, and encourage the use of shared molecular data across *Prunus* species. The curated data includes 177 genetic maps, primarily for almond (19), apricot (21), peach (52), and sweet cherry (46). A total of 28 971 trait-associated loci were reported, with 72.4% derived from genome-wide association studies, 18.7% from quantitative trait loci (QTL), and 8.9% from Mendelian trait loci. Notably, 76.4% of these loci are associated with morphological and quality traits, reflecting breeders’ focus on consumer preferences. We identified 16 potential QTL hotspots linked to key traits such as morphology, phenology, fruit quality, and disease resistance. Additionally, we identified 17 high-priority syntenic regions among peach, sweet cherry, and almond. The colocalized markers and genes within the QTL hotspots and syntenic regions offer a valuable resource for tool development for *Prunus* breeding, especially for complex polyploid genomes and lesser studied species with limited genetic and genomic data.

## Introduction

Recent advances in molecular technology have expanded our understanding of plant genetics. These strides have improved several key approaches in plant breeding including analyses of genetic variability, creation and application of genetic markers, construction and interpretation of high-density linkage maps, identification of marker–trait associations through genome-wide association studies (GWAS), and the integration of marker-assisted selection in breeding programs. Furthermore, these advancements have fostered the emergence of the field of genetical genomics, which combines high-throughput ‘omics’ data (such as expression or genomic data) with genetic information [1, 2]. The large volume of genetic, genomic, expression, and breeding data generated necessitates sophisticated management, storage, and analysis methods. To address these needs, advanced databases such as the Genome Database for Rosaceae (GDR, https://www.rosaceae.org), have been developed. GDR serves as a comprehensive repository for *Rosaceae* family, including *Prunus* species, thereby supporting both research and applied breeding efforts [3]. Since the review of Salazar *et al*. [4] on curated *Prunus* quantitative trait loci (QTL) and Mendelian trait loci (MTL) data housed in the GDR, there has been a significant surge in genetic and genomic research on *Prunus* species, driven in part by the growing accessibility and affordability of high-throughput genotyping technologies. As a result, a wealth of genetic and genomic information is embedded within the extensive molecular data published in the literature.

*Prunus* species are among the most extensively studied plants within the Rosaceae family. *Prunus* species are commercially significant sources of fruit, nuts, and ornamental trees. Key species include almonds (*Prunus dulcis*), apricots (*Prunus armeniaca*), cherries (*Prunus avium* and *Prunus cerasus*), peaches (*Prunus persica*), and plums (*Prunus domestica* and *Prunus salicina*) [5]. Globally, *Prunus* species have an annual fruit production of ~50 million tons across 7.7 million ha, and the most cultivated are peach (26.4 million tons in 1.5 million ha), plums (12.4 million tons in in 2.6 million ha), apricot (3.8 million tons in 0.5 million ha), almond (3.6 million tons in 2.3 million ha), sweet cherry (2.7 million tons in 0.4 million ha), and sour cherry (1.6 million tons in 0.2 million ha) [6]. A major component of the extensive studies conducted on *Prunus* species are genetic and genomic analyses which include QTL/MTL mapping and GWAS.

**Table 1:** Total number of genetic maps developed for *Prunus* species between 1998 and 2023.

**s/n**	**Species**	**Common name**	**No. of maps**	**% of maps**	**Pop. Size**	**No. of LGs**	**No. of loci**
1	*P. armeniaca*	Apricot	21	11.9	113	7.8	69
2	*P. avium*	Sweet cherry	46	26.0	132	8.6	966
3	*P. cerasifera*	Cherry plum	1	0.6	NA	1	2
4	*P. cerasus*	sour cherry	9	5.1	74	8.9	1519
5	*P. davidiana*	David’s peach	11	6.2	131	6.3	64
6	*P. dulcis*	Almond	19	10.7	88	8.2	522
7	*P. mume*	Japanese apricot	2	1.1	289	8	4075
8	*P. persica*	Peach	52^a^	29.4	115	8	327
9	*P. prunus*	Plums	8	4.5	111	7.2	459
10	*P. salicina*	Japanese plum	2	1.1	151	8	563
11	*P. spachiana*	N/A	1	0.6	178	6	17
12	*P. virginiana*	chokecherry	2	1.1	101	29	563
13	*P. spp*	*Prunus* hybrids	3	1.7	178	4	27

a Four maps of *P. ferganensis* are listed under *P. persica* following reclassification [23, 24].

Several QTL/MTL studies conducted on *Prunus* species resulted in the mapping of key traits and the identification of QTL hotspots [7–12]. QTL hotspots are regions enriched in QTLs, statistically harboring a significantly higher number of QTLs than expected by random chance, and are often responsible for correlated traits [13, 14]. Some of the possible causes of the QTL hotspot phenomenon are allelic polymorphism and pleiotropy. QTLs with high allelic polymorphisms have a high chance of detection across different studies, and pleiotropic or closely linked QTLs controlling correlated traits are often colocalized [15]. Similarly, several genetic studies on *Prunus* species report trait-associated regions with conserved gene content known as syntenic regions [16–19]. Although QTL hotspots and syntenic regions are well documented in *Prunus* species, there is limited understanding regarding the transferability of QTL, MTL, or GWAS data between different *Prunus* species, especially from well-studied to less-studied species. Aranzana *et al*. [20] recently reviewed key achievements in *Prunus* genetics and offered insights into the evolutionary dynamics of *Prunus* genomes. In line with this, a comprehensive analysis of the genetic architecture of the *Prunus* genus would expand breeding options and facilitate the sharing of trait locus information among its various species.

We recently made a major effort to enhance the GDR with updated QTL and MTL data, along with quantitative trait nucleotides (QTNs derived from GWAS). Early genetic studies primarily linked QTLs to simple sequence repeat (SSR) markers with no reported genome positions. However, with the advent of high-throughput genotyping and sequencing technologies, which have led to the availability of multiple whole-genome assemblies for *Prunus* species, recent studies increasingly associate QTLs with single nucleotide polymorphism (SNP)-based markers mapped to specific genome positions (physical positions in base pairs). In our work, we have aligned a variety of SSR markers and their associated QTLs across different *Prunus* genomes. The alignments were made using curated data from >200 peer-reviewed publications within the GDR. This report provides a comprehensive summary of *Prunus* QTLs, MTLs, and QTNs reported during a 25-year period from 1998 to 2023. It aims to (1) explore the comprehensive genetic architecture underlying key traits in *Prunus*, (2) provide ready-to-use data for crop improvement, and (3) promote the use of shared molecular data, especially from QTL hotspots and syntenic regions, across *Prunus* species to enhance breeding efforts.

## Results

### Curated data in GDR

A total of 177 genetic maps of various *Prunus* species, developed between 1998 and 2023, were downloaded from the GDR, and the average population size, number of linkage groups, and number of loci per *Prunus* species were calculated (Table 1). Among these, peach [25], sweet cherry [26], apricot [17], and almond [27] had the highest numbers of genetic maps compared to other *Prunus* species (Table 1; www.rosaceae.org/tripal_megasearch). Most genetic maps in sweet cherry, apricot, and almond were developed through intraspecific crosses, pointing to self-incompatibility in these species. These genetic maps are the source of the reported QTLs and MTLs during the period under consideration (1998–2023).

QTLs, MTLs, and QTNs linked to several agronomic traits in *Prunus* species are stored in the GDR. A total of 28 971 trait-associated loci were reported between 1998 and 2023, of which 72.4% were QTNs, 18.7% were QTLs, and 8.9% were MTLs (Table 2). The most studied traits in *Prunus* species were morphological and quality traits accounting for 76.4% of the reported genomic regions, with morphological traits alone accounting for 52.2%. The stress and fertility traits had the lowest number (2.3%) of the reported genomic regions in *Prunus* species (Table 2).

**Table 2:** Total number of genomic regions in *Prunus* species reported between 1998 and 2023.

**Trait category**	**QTL (%)**	**MTL (%)**	**QTN (%)**	**Sum**	**% in all**
Morphology	223 (1.5)	4275 (28.3)	10 619 (70.2)	15 117	52.2
Quality	353 (5)	972 (13.9)	5673 (81.1)	6998	24.2
Yield	230 (6)	NA	3599 (94)	3829	13.2
Growth and dev.	999 (67.5)	30 (2)	452 (30.5)	1481	5.1
Biochemical	426 (48.5)	NA	453 (51.5)	879	3.0
Stress	350 (58.7)	88 (14.8)	158 (26.5)	596	2.1
Fertility	6 (8.5)	54 (76)	11 (15.5)	71	0.2

A total of 879 genomic regions were reported to regulate several biochemical traits including enzyme activities, metabolite contents, pH, and taste, among which 429 were QTLs and 453 were QTNs (Table 2, www.rosaceae.org/tripal_megasearch). A total of 6998 genomic regions were reported to regulate quality traits in *Prunus* species including blush coverage, tissue/organ colors, skin toughness, flesh adhesion, tissue/organ size and dimensions, etc. (www.rosaceae.org/tripal_megasearch). Among these, 5% were QTLs, 13.9% were MTLs, and 81.1% were QTNs (Table 2). The growth & development traits in *Prunus* species are regulated by 1481 genomic regions of which 999 are QTLs, 30 MTLs, and 452 QTNs. These growth and development traits include phenological traits, chilling requirement, internode length, and growth habits. A total of 3829 genomic regions regulate yield traits in *Prunus* species, of which 6% are QTLs, and 94% are QTNs. The yield-related traits recorded within the past 25 years were mainly weight of fruit and fruit parts (www.rosaceae.org/tripal_megasearch). Morphological traits in *Prunus* species are regulated by 15 117 genomic regions of which 1.5% were QTLs, 28.3% were MTLs, and 70.2% were QTNs. These include organ shapes and dimensions, shell hardiness, fruit flesh adhesion, etc.. The stress traits studied in *Prunus* species were mainly disease resistance and resistance to parasitic worms. A total of 596 genomic regions regulating plant response or resistance to these biotic stresses have been identified, among which 58.7% are QTLs, 14.8% are MTLs, and 26.5% are QTNs. A few genomic regions control fertility traits such as gametophytic incompatibility, pollen germination, and pollen fertility, with 8.5% of the genomic regions being QTLs, 76% MTLs, and 15.5% QTNs (www.rosaceae.org/tripal_megasearch). Overall, several QTLs, MTLs, and QTNs have been identified for most trait categories, but no MTLs were reported for yield and biochemical traits, highlighting their polygenic nature (Table 2).

### QTL and MTL numbers after alignment to *Prunus* genomes

The initial counts of QTL and MTL across the four *Prunus* genomes (*P. persica* v2, *P. avium* Tieton v2.0, *P. avium* Regina v1.0, and *P. dulcis* Lauranne v1.0) were 84, 0, 133, and 0, respectively. Upon subsequent alignment of markers to the *Prunus* genomes, the number of QTLs/MTLs increased to 440, 57, 190, and 71, for *P. persica* v2, *P. avium* Tieton v2.0, *P. avium* Regina v1.0, and *P. dulcis* Lauranne v1.0, respectively, highlighting the effectiveness of the marker alignments.

### QTL hotspots in Prunus

A total of 16 QTL hotspots were identified in the four genomes, with most of the hotspots found in peach and almond genomes. In linkage group (LG) 1, four hotspots (LG1–1, LG1–2, LG1–3, and LG1–4) were identified. LG1–1 (9 299 688..0.929919 Mb) was found in apricot and is involved in resistance to plum pox virus. LG1–2 and LG1–3 were found in peach and sweet cherry and mainly contain QTLs for fruit morphological and growth & development (shelf life-related) traits. LG1–4 was found in peach and contains QTLs for 50% bloom date and organic acids (Table 3). Three QTL hotspots (LG2–1, LG2–2, and LG2–3) were identified in LG2 containing QTLs for biochemical, growth and development (phenological), morphological, and disease resistance traits. QTLs from peach were identified in all three hotspots, whereas sour cherry had a QTL for 50% bloom date within the hotspot region LG2–2. In LG3, three QTL hotspots (LG3–1, LG3–2, and LG3–3) were identified. The hotspot region LG3–1 contains QTLs for shelf life-related traits, phenological traits, resistance to bacterial spot (*Xanthomonas arboricola* pv. *pruni*), and powdery mildew (*Podosphaera pannosa*). LG3–2 contains QTLs for biochemical and fruit color traits, whereas LG3–3 contains fruit morphological (shape and size) traits.

**Table 3:** QTL hotspots for fruit traits in *Prunus* species aligned to the Peach v2 genome [28].

**Trait name**	**Organism**	**LG**	**Region (bp)**	**Region (cM)**	**Map**	**Colocalizing marker**	**Population**	**References**
Resistance to plum pox virus, fruit length; thickness; width; fresh weight; resistance to *M. fructicola*; cracking; firmness	*P. armeniaca;* *P. persica; P. avium*	LG1–1	9299688..26400000	33..70 (*P. armeniaca*); 28.25..96.29 (*P. persica, P. avium*); 104.76..120.38 (*P. avium*)	Apricot-LL-F2-Lito-2011 Peach-WB-F2–2015; Peach_CxEL_F1; Peach_CxEL_F1-physical-*Prunus*-persicaV1.0; Sweet Cherry-RosBREED-1.6 K-2019; Sweet Cherry-A-F1; Sweet_Cherry-Crump-panel-2022	Gol027; RosBREED_snp_sweet_cherry_Pp1_12191200; RosBREED_snp_sweet_cherry_Pp1_14281912; RosBREED_snp_sweet_cherry_Pp1_11060284; RosBREED_snp_sweet_cherry_Pp1_11107790; SNP_IGA_31108; SNP_IGA_32535; SNP_IGA_37651; RosBREED_snp_sweet_cherry_Pp1_15061804; RosBREED_snp_sweet_cherry_Pp1_13761187; RosBREED_snp_sweet_cherry_Pp1_14110306; RosCOS1084-357_snp_sweet_cherry_Pp1_16608849; RosBREED_snp_sweet_cherry_Pp1_14779305	Lito_x_Lito-98-F2; NJ_Weeping_x_Bounty; Contender_x_ElegantLady_F1; Ambrunes_x_Sweetheart; Crump-panel-2022	[19, 29] [30–33]
Bloom date 50%; chilling requirement; fruit length; fresh weight, width, thickness	*P. persica*	LG1–2	41168691.. 43578596	83..101.12	Peach-CF-F2; Peach-HU-F2	CPPCT029; SNP_IGA_121865; pchgms29	Contender_x_Fla.92–2C-F2; Hakuho_x_UFGold; NJ_Weeping_x_Bounty	[31, 34–36]
Bloom date 50%; p-coumaric acid; p-coumaroyl quinic acid; neochlorogenic acid	*P. persica; P. avium*	LG1–3	44 069 867.. 47580000	141.34..182	Peach-RosBREED-4 K-2019; Sweet Cherry-V-F1–6 + 9K_array	SNP_IGA_133606; SNP_IGA_132901; scaffold_1:42343512; s1_42801165	Vic_x_Cristobalina	[37, 38]
Fruit length; bloom date 50%	*P. persica*	LG2–1	13486292.. 13651450	11.39..29	Peach-WB-F2–2015; Peach-CF-F2–2014	SNP_IGA_240162; UDP-025	NJ_Weeping_x_Bounty; Contender_x_Fla.92–2C-F2	[31]
Thickness; resistance to chilling injury; resistance to *M. fructicola*; width; bloom date 50%	*P. persica; P. cerasus*	LG2–2	17410079.. 19354729	17.911..24.483 (*P. persica*); 20.9..43 (*P. cerasus*).	Peach-WB-F2–2015; Peach-VV-F2; Peach_CxEL_F1; Peach_CxEL_F1-physical-*Prunus*-persicaV1.0; Sour Cherry-US-F1	SNP_IGA_259667; SNP_IGA_262277; BPPCT004; SNP_IGA_263828; sweet_cherry_Pp2_15778222	NJ_Weeping_x_Bounty; Venus_ x_Venus-F2; Contender_x_ElegantLady_F1; Ujfehertoi_Furtos_x_Surefire	[31, 39]
p-coumaric acid; p-coumaroyl quinic acid; fresh weight	*P. persica*	LG2–3	2240000.. 7217184	3.85..5.02	Sweet Cherry-C-F1–6 + 9K_array; Peach-WB-F2–2015	sweet_cherry_Pp2_02274343; SNP_IGA_170137	Vic_x_Cristobalina; NJ_Weeping_x_Bounty	[31, 37]

(Continued)

**Table 3:** Continued

**Trait name**	**Organism**	**LG**	**Region (bp)**	**Region (cM)**	**Map**	**Colocalizing marker**	**Population**	**References**
Fruit hardness; resilience; loss of firmness; cohesiveness; resistance to *X. arboricola* pv. pruni; resistance to powdery mildew; soluble solids content; skin overcolor; fresh weight; bloom date 50% (*P. cerasus*); development period; ripe 100%; bloom date 50%	*P. persica; P. cerasus*	LG3–1	10057674.. 13633831	0..36.60	Peach-BA-F1–2017; Peach-RosBREED-4 K-2019; Peach-Nectaross-F1; Peach-BN-F1–2017; Peach-Armking-F1; Peach-WB-F2–2015; Peach-VV-F2; Peach_CxEL_F1; Peach_CxEL_F1-physical-*Prunus*-persicaV1.0; Sour Cherry-US-F1; Peach-OC-F2; Peach-SP-BC2–2003; Peach-VV-F2; Peach-CF-F2–2014	SNP_IGA_407919; SNP_IGA_407919; SNP_IGA_408505; SNP_IGA_408884; SNP_IGA_409274; SNP_IGA_410165; SNP_IGA_410336; SNP_IGA_410398; EPPCU9268; tart_cherry_f_Pp4_10832168; SNP_IGA_410794; SNP_IGA_411601; CC138; CBV_ANAC072; Pchgms167; SNP_IGA_415301	Bigtop_x_Armking; Bigtop_x_Nectaross; NJ_Weeping_x_Bounty; Venus_x_Venus-F2; Bigtop_x_Nectaross; Contender_x_ElegantLady_F1; Ujfehertoi_Furtos_x_Surefire; Ohenry_x_Clayton-F2; Summergrand_x_P1908-BC2; Venus_x_Venus-F2; Contender_x_Fla.92–2C-F2	[12, 25, 26, 30, 35, 38, 39, 42, 40, 41]
Peonidin 3-O-glucoside; cyanidin 3-O-glucoside; flesh color; skin color	*P. avium*	LG3–2	15750000.. 18510000	9.52..86.37	Sweet Cherry-C-F1–6 + 9K_array	sweet_cherry_Pp3_12070148; sweet_cherry_Pp3_12987920; sweet_cherry_Pp3_13520194; sweet_cherry_Pp3_13025963; scaffold_3:9917277	Vic_x_Cristobalina	[37]
Fruit length; firmness; width; fresh weight	*P. persica; P. avium*	LG3–3	18179421.. 22294942	37.82..66	Peach-WB-F2–2015; Sweet Cherry-RosBREED-1.6 K-2019; Sweet_Cherry-Crump-panel-2022	SNP_IGA_341962; sweet_cherry_Pp3_15682037; sweet_cherry_Pp3_15682037; sweet_cherry_Pp3_13290870; SNP_IGA_344628	NJ_Weeping_x_Bounty; Crump-panel-2022	[31, 33]
Skin overcolor; fruit fresh weight; resistance to chilling injury; bloom date 50%; fruit development period; fruit ripe 100%	*P. persica*	LG4	10641209.. 13633831	12.00..49.00	Peach-RosBREED-4 K-2019; Peach-WB-F2–2015; Peach-VV-F2; Peach-BN-F1–2017; Peach_CxEL_F1; Peach_CxEL_F1-physical-*Prunus*-persicaV1.0; Sour Cherry-US-F1; Peach-OC-F2	SNP_IGA_410165; SNP_IGA_410336; SNP_IGA_410398; EPPCU9268; tart_cherry_f_Pp4_10832168; SNP_IGA_410794; SNP_IGA_411340; SNP_IGA_411601	NJ_Weeping_x_Bounty; Venus_x_Venus-F2; Bigtop_x_Nectaross; Contender_x_ElegantLady_F1; Ujfehertoi_Furtos_x_Surefire; Ohenry_x_Clayton-F2	[12, 25, 26, 30, 31, 39, 41, 42]
Time to maturity; bloom date 50%; chilling requirement; fruit cracking; resistance to *X. arboricola* pv. pruni	*P. persica; P. cerasus*	LG5–1	10 338 702.. 11947495	11.6..34.4 (*P. cerasus*); 2.3..56 (*P. persica*)	Peach-Bigtop-F1; Sour Cherry-M172x25-F1; Peach-CF-F2–2014; Sweet Cherry-RosBREED-1.6 K-2019; Peach-OC-F2	SNP_IGA_588670; sweet_cherry_Pp5_10500237; ssrPaCITA21; s5_15123033; SNP_IGA_594090	M172_x_25–02–29; Contender_x_Fla.92–2C-F2	[25, 34, 35, 42, 43, 33]

(Continued)

**Table 3:** Continued

**Trait name**	**Organism**	**LG**	**Region (bp)**	**Region (cM)**	**Map**	**Colocalizing marker**	**Population**	**References**
Total water-soluble content; fruit cracking; titratable acidity; resistance to *M. fructicola*; resistance to powdery mildew; resistance to *Dysaphis cf. devecta*	*P. persica; P. avium*	LG5–2	15249344.. 19450969	31..53 (*P. avium*) 58..72 (*P. persica*)	Peach-RosBREED-4 K-2019; Sweet_Cherry-Crump-panel-2022; Peach_CxEL_F1; Peach-SP-F2–2003; Peach-SP-F1–1998	snp_5_15254637; RosCOS1765-517_snp_sweet_cherry_Pp5_15546701; SNP_IGA_602331; SNP_IGA_615381; UDP-407; AG26	Crump-panel-2022; Contender_x_ElegantLady_F1; Summergrand_x_P1908-F2	[26, 30, 33, 44, 40]
Titratable acidity; total water-soluble content; fruit cracking; resistance to *M. fructicola*; resistance to powdery mildew; resistance to *D.* cf. *devecta*	*P. persica; P. avium*	LG6–1	1503387.. 19450969	2..8; 58..72	Peach-RosBREED-4 K-2019; Sweet_Cherry-Crump-panel-2022; Peach_CxEL_F1; Peach-SP-F2–2003; Peach-SP-F1–1998	SNP_IGA_548512; snp_5_15254637; RosCOS1765-517_snp_sweet_cherry_Pp5_15546701; SNP_IGA_602331; SNP_IGA_615381; UDP-407; AG26	Crump-panel-2022; Contender_x_ElegantLady_F1; Summergrand_x_P1908-F2; Summergrand_x_P1908-F1	[30, 33, 40, 44, 26]
Time to maturity; resistance to *M. fructicola*; fruit hardness; quinic acid content; fruit thickness; fruit width	*P. persica*	LG6–2	3937990.. 4823411	1.72..2.58	Peach-BA-F1–2017; Peach_CxEL_F1; Peach_CxEL_F1-physical-*Prunus*-persicaV1.0; Peach-Armking-F1; Peach-BA-F1–2017; Peach-JF-F2; Peach-WB-F2–2015	SNP_IGA_604703; SNP_IGA_605027; FG215; SNP_IGA_620099	Bigtop_x_Armking; Contender_x_ElegantLady_F1; Ferjalou_Jalousia_x_Fantasia-F2; NJ_Weeping_x_Bounty	[25, 31, 30, 45]
Bloom date 50%; chilling requirement	*P. persica*	LG7	15784304.. 16365104	24..70	Peach-RosBREED-4 K-2019; Peach-CF-F2–2014; Peach-CF-F2; Peach-HU-F2	SNP_IGA_779362; Pchgms226; UDAp-460; CPPCT033; SNP_IGA_780816	Contender_x_Fla.92–2C-F2; Hakuho_x_UFGold	[34, 35, 36, 38]
Chilling requirement; growing degree days for ecodormancy release	*P. persica*	LG8	18135154.. 18135253	24..54	Peach-CF-F2; Peach-CF-F2–2014	PacC13	Contender_x_Fla.92–2C-F2	[34, 35]

LG4 harbors one QTL hotspot in peach (10 641 209..0.13633831 Mb) with various QTLs (Table 3). Two hotspots (LG5–1 and LG5–2) exist in LG5. LG5–1 contains QTLs for phenological traits, fruit cracking, and resistance to bacterial spot. LG5–2 contains QTLs mainly controlling water-soluble content, titratable acidity, and resistance to brown rot (*Monilinia fructicola)*, powdery mildew, and leaf curl (*Taphrina deformans*). Peach had QTLs in both hotspots, whereas sour cherry and sweet cherry each had a QTL in one hotspot in LG5. Two QTL hotspots (LG6–1 and LG6–2) are found in LG6, and they contain various QTLs including biochemical (titratable acidity, water-soluble content), morphology and shelf life-related traits (fruit cracking, fruit hardness, fruit thickness, width), and disease-resistance traits (resistance to brown rot, powdery mildew, and leaf curl). Peach had QTLs in both hotspots on LG6, whereas sweet cherry had QTLs in one hotspot (LG6–1). LG7 and LG8 each had one QTL hotspot both containing QTLs for phenological traits including bloom date 50% (in LG7 alone); chilling requirement, and growing degree days for ecodormancy release (in LG8 alone). Both hotspots contain only peach QTLs.

### QTLs conserved across *Prunus*

**Figure 1:**
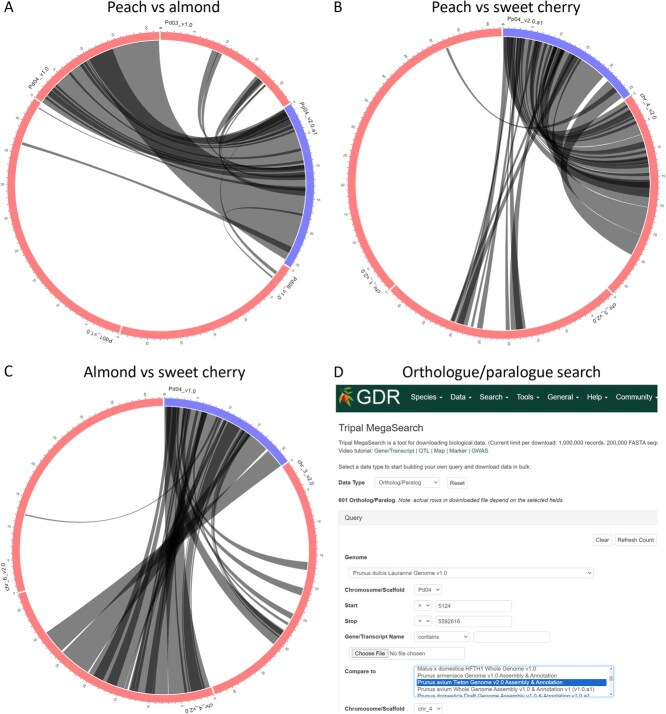
Chr 4 syntenic relationships in *Prunus*. Synteny between (A) peach and almond, (B) peach and sweet cherry, (C) almond and sweet cherry, and (D) an example of orthologue/paralogue search using the genome positions obtained from the synteny analysis of almond versus sweet cherry. Blue lines represent selected chromosomes; red lines are chromosomes from the genome being compared to; black lines are syntenic blocks.

Seventeen syntenic regions were identified across peach, sweet cherry, and almond genomes (Table 4). All chromosomes (Chr), except Chr 3, harbor at least one syntenic region. Four syntenic regions were found on Chr 7, and three each on Chr 4 and 6. All syntenic regions control a single disease resistance trait (powdery mildew, plum pox virus, or brown rot) and/or phenological traits (chilling requirement, fruit ripe 100%, bloom date 100%, and growing degree days for ecodormancy release), suggesting adaptation to these biotic and abiotic stresses. For each syntenic region, the names of QTL/MTL, location in each of the three *Prunus* genomes, colocalizing SSR markers, and primers are shown (Table 4). Further analysis of the syntenic regions on Chr 4 showed syntenic blocks between each crop pair (Fig. 1; Tables S1, S3, and  S5). The peach/almond, peach/sweet cherry, and almond/sweet cherry pairs contain syntenic blocks pdlppB101 and pdlppB102, ppptB166 and pdlptB128, respectively within 5–25 Mb. Key genes within the syntenic blocks are provided in Tables S2, S4, and  S6.

**Table 4:** Syntenic regions that contain QTLs in *Prunus.*

**Trait**	**LG**	**QTL label**	**Region in peach (bp)**	**Region in sweet cherry (bp)**	**Region in almond (bp)**	**Colocalized marker**	**Primers**	**References**
Res. to powdery mildew	LG1	qPMR.SP-ch1.1-SD402Not5	1298857..1299112	1447378..1447618 (Regina); 5654117..5654357 (Tieton)	18252132..18252360	UDP-018	F: TTCTAATCTGGGCTATGGCG; R: GAAGTTCACATTTACGACAGGG	[40, 46]
Res. to plum pox virus	LG1	qRPPV.LL-Lito-LG1	9299688..9299919	NA	8708884..8709125	Gol027	F: TGCACTGTCAACCATGTCTTTT; R: TGGTACTGAGACTGCTGACAGA	[19]
Res. to powdery mildew	LG2	qPMR.SP-ch2-SD402Not9a	24830251..24830441	33005284..33005567 (Regina); 38694995..38695318 (Tieton)	19408865..19409052	pchgms1	F: GGGTAAATATGCCCATTGTGCAATC; R: GGATCATTGAACTACGTCAATCCTC	[47, 40]
Chilling requirement; res. to *M. fructicola*	LG4	qCR.27.2.CxF-G4b.2009	9219594..9220930	11995736..11995911 (Regina); 13179486..13179661 (Tieton)	9584452..9584632	M12a	F: AGGTGCCTCATCTTCTTCTCTTG; R: GTGTGGTGAGGGGTGAGAGC	[30, 34, 35, 48]
Fruit ripe 100%	LG4	qRPT.VV-Ch4–2012	11116962..11117853	14259517..14260400 (Regina); 15991138..15992022 (Tieton)	11468231..11469123	CBV_ANAC072	F: ATGGGTGTGCCAGAAACCGACCCA; R: CCGAGCTTGCTGTCCTCCTGCT	[39]
Bloom date 50%	LG4	qBD.CxF-G4b.2008.2014	12441742..12441991	NA	12474679..12474904	Pchgms167	F: TCAATGCTTATGCTTGCTTG; R: AATGAATATCCACCAAATAGACTG	[35]
Res. to *M. fructicola*	LG5	qRMF.CxEL-LG5.2009.FL-if	12306819..12306963	13806107..13806247 (Regina); 27719283..27719423 (Tieton)	11152293..11152427	BPPCT037	F: CATGGAAGAGGATCAAGTGC; R: CTTGAAGGTAGTGCCAAAGC	[27, 30]
Bloom date 50%	LG5	qBD.CxF-G5.2008	10776260..10776499	NA	9617572..9617808	ssrPaCITA21	F: GATTATATAAGTTGGTTTTTGTAAG; R: GTATTCTATAATGTATAAATGTACG	[49]
Res. to *M. fructicola*	LG6	qRMF.CxEL-LG6.2010.Sk-if	26722120..26722393	29618835..29619059 (Regina); 33081125..33081375 (Tieton)	24532488..24532726	MA040a	F: AGAAATTGGAGTGACGTAAC; R: ACGTGATGAGAAGTAGGGAG	[18, 29, 30]
Res. to powdery mildew	LG6	qPMR.SP-ch6-SDNot1	21030767..21031014	34251382..34251630 (Regina); 26576858..26577106 (Tieton)	NA	pchcms5	F: CGC CCA TGA CAA ACT TA; R: GTC AAG AGG TAC ACC AG	[40, 47]
Chilling requirement	LG6	qCR.2.CxF-G6.2008	28325429..28325641	NA	26191808..26192017	EPPISF002	F: CGACGTGTGACCAAAGGAC; R: GCAACTCCATCCACATTTCTC	[50]

(Continued)

**Table 4:** Syntenic regions that contain QTLs in *Prunus.*

**Trait**	**LG**	**QTL label**	**Region in peach (bp)**	**Region in sweet cherry (bp)**	**Region in almond (bp)**	**Colocalized marker**	**Primers**	**References**
Res. to *M. fructicola*	LG7	qRMF.CxEL-LG7.2009.Sk-rd.3	21384787..21385070	28490554..28490676 (Regina); 29587313..29587435 (Tieton)	NA	UDAp-407	F: TTCTGCTACTTACAATCGTGTTCTC; R: AGAGCACCAGGTCTTTCTGG	[17, 30]
Res. to *M. fructicola*	LG7	qRMF.CxEL-LG7.2009.Sk-rd.2	18371394..18371933	25244092..25244250 (Regina); 26246465..26246623 (Tieton)	NA	EPPCU5176	F: ATGACCACACAGAATCACCC; R: GATCCTCAGCCCGAGTCAAT	[29, 30]
Bloom date 50%; chilling requirement	LG7	qBD.CxF-G7a.2007.2014	16270845..16270995	22827978..22828134 (Regina); 23631889..23632045 (Tieton)	13664991..13665123	UDAp-460	F: TCATCAGTCAGGTGGTGCTC; R: TGACAGCCTAATCAGCCATTT	[51]
Res. to powdery mildew	LG7	qPMR.SP-ch7-SD402Not6	19089997..19089997 (Ppv1)	NA	16381178..16381357	pchcms2	F: AGGGTCGTCTCTTTGAC; R: CTTCGTTTCAAGGCCTG	[40, 52]
Chilling requirement; growing degree days for ecodormancy release	LG8	qCR.2.CxF-G8.2008	18135154..18135253	21554007..21554118 (Regina); 31856407..31856518 (Tieton)	14805668..14805767	PacC13	F: GCTTGCTGCTCATCATTTAC; R: AATAACAACCATATTGGAGTATTTAC	[34, 35, 53]
Chilling requirement	LG8	qCR.2.CxF-G8.2008	18135154..18135253	21554007..21554118 (Regina); 31856407..31856518 (Tieton)	14805668..14805767	PacC13	F: GCTTGCTGCTCATCATTTAC; R: AATAACAACCATATTGGAGTATTTAC	[34, 53]

## Discussion

Modern plant breeding initiatives produce exceptionally large volumes of genetic, genomic, and phenotypic data, which are essential for identifying key genomic regions. The effective management, storage, and analysis of these data are largely dependent on proficient use of genomic databases. The GDR, first established in 2003, is a repository for genetic, genomics, and phenotypic data for Rosaceae family crops from genera such as *Fragaria, Malus, Prunus, Pyrus, Rosa,* and *Rubus*, among others [3]. Data from multiple studies on Rosaceous species have been systematically curated into the GDR. In this study, we employed the data housed in the GDR to examine the genetic architecture of key traits in genus *Prunus*. We investigated the potential for utilizing shared genetic markers to study multiple *Prunus* species, particularly when markers or genes are located within the same QTL hotspot or syntenic block.

*Prunus* species have garnered substantial research attention within the Rosaceae family due to their economic importance. During the past 25 years, genetic studies have developed >177 *Prunus* genetic maps of which 29.4% were for peach, 26% for sweet cherry, 11.9% for apricot, and 1.7% were for almond. Most of the research focused on fruit quality and morphological traits, which together account for 76.4% of all reported genomic regions, underscoring their economic value and the need to meet consumer preferences. Fruit quality and morphological trait categories have high number of MTLs compared to other trait categories, indicating the presence of individual monogenetic traits. These traits include anther color, flesh color, fruit shape, fruit skin color, skin pubescence, leaf shape, etc., which exhibit Mendelian inheritance patterns. Conversely, biochemical and yield traits have no MTLs in GDR, indicating the absence of monogenetic traits and the presence of polygenic traits that have quantitative inheritance patterns requiring the contribution of many genes [54].

### Potential QTL hotspots for enhanced breeding in *Prunus*

Most QTLs/MTLs within the 16 potential QTL hotspot regions determined in this study are related to fruit morphology, growth and development (phenology) traits, and quality traits such as skin color and shelf life-related traits, further highlighting the key breeding interest in *Prunus* species. The higher number of peach QTLs observed in the hotspots compared to other *Prunus* species reflects the extensive research focus on peach. This is partly attributed to the simplicity of the peach genome (diploid, ~230 Mb), short juvenility period (<4 years), and the availability of valuable genetic resources, such as the peach × almond (TxE) progeny developed in the 1990s [22, 55]. In the current study, due to the large numbers of QTNs obtained from GWAS, we have included only QTLs and MTLs in the hotspots, with ready-to-use marker information for breeders. Detailed QTN information can be obtained from the Megasearch tab in GDR. Our approach to determining potential QTL hotspots by considering the physical positions of the QTLs within 4 Mb of each genome is a conservative estimate. QTL hotspots can range in size from compact to more extended genomic segments. For instance, Kale *et al*. [56] reported a QTL hotspot region for drought tolerance in chickpea (*Cicer arietinum* L.) spanning 29 cM, corresponding to ~7.74 Mb on the physical map, while Boopathi *et al*. [57] identified a QTL hotspot with a span length of 89.4 cM in cotton (*Gossypium hirsutum*). In peach and sweet cherry, QTL hotspots ~1–4 Mb in length were reported on Chr 4 [11, 58]. The variation in hotspot sizes underscores their dynamic nature and suggests that QTL hotspots can encompass a wide range of genetic distances influenced by factors such as trait complexity, genetic interactions, population dynamics, and analytical method [14, 59]. Previous studies have shown the limitations of using permutation algorithms such as the quantile-based permutation, which may result in excessive detection of spurious hotspots and failure to discover biologically interesting hotspots that harbor a small to moderate number of QTLs [14, 43]. In the current study, algorithm-based QTL hotspot detection was not conducted. Instead, a systematic search of the physical locations (in base pairs) of the QTLs was done using data from GDR (Table 3). This method would require further validation using statistical methods. Li *et al*. [60] investigated linkage disequilibrium (LD) decay across various oriental and occidental peach populations. Their findings revealed that, in most populations, LD decay occurred within a range of 1.9–3.15 Mb (3.36–6.3 cM). However, in a nested subpopulation of occidental peaches, LD decay extended to as far as 12.86 Mb (24.93 cM). Similarly, LD decay in various sweet cherry populations—including wild cherry, landraces, and modern cultivars—ranged from as little as 1 cM to as much as 19 cM [21]. These findings further highlight the potential variation in QTL hotspot regions and suggest that candidate genes located within the LD decay distance are valuable targets for *Prunus* crop improvement. Overall, the colocalizing markers (Table 3) and candidate genes linked to each potential hotspot could be a good resource for marker-assisted breeding in *Prunus*. Users can utilize the Gene Search tool in the GDR to retrieve gene lists and functional annotations based on genomic location. In addition, the Marker Search tool allows users to identify markers located within these hotspots, facilitating further trait-specific analyses and fine-mapping efforts.

### Syntenic regions to guide breeding decisions in *Prunus*

Even though the importance of syntenic regions in plant breeding has been extensively reviewed [20, 61–64], the application of synteny information in crop breeding remains limited. This limitation is partly because many studies do not focus on specific syntenic blocks nor provide readily usable information, such as markers and primers, which are essential for practical breeding applications. In the current study, we identified 17 syntenic regions with each showing colocalized markers and primers (Table 4). For example, the syntenic regions on Chr 4 (0.6–19.0 Mb) between peach, almond, and sweet cherry (Fig. 1) contain numerous QTLs, particularly concentrated between 9.0 and 12.5 Mb. This hotspot region is associated with various traits, including bloom date, fruit weight, pit roundness, flesh adhesion, ripening date, fruit firmness, and water-soluble content [11]. The fruit quality and disease resistance QTLs clustered in this syntenic hotspot have been recently reviewed [58]. Further synteny information for other chromosomes and *Prunus* genomes can be found on the Synteny Viewer in GDR (https://www.rosaceae.org/synview/search).

The compact *Prunus* genome (<300 Mb) makes it an excellent reference for identifying markers in related species [16, 24, 28]. The synteny data presented in this study could be particularly valuable for orphan *Prunus* crops and other genera within the Rosaceae family. Dirlewanger *et al*. [16] conducted a comparative genomic analysis on seven diploid *Prunus* species, including almond, peach, cherry, and apricot, using a consensus *Prunus* map. Their findings revealed marker collinearity and syntenic genes, which underscored the crossability patterns observed among *Prunus* species and suggested that the *Prunus* genus can be regarded as a single genetic entity at the genomic level. Moreover, whole-genome comparisons of *Prunus*, apple (*Malus*), and strawberry (*Fragaria*) have shown that *Prunus* has the most conserved karyotype at both the macro- and microsyntenic level in relation to the ancestral genome configuration for Rosaceae [65]. Similarly, Dondini *et al*. [17] compared apricot maps with published *Prunus* maps including those of peach × almond, myrobalan × (almond × peach), and (peach × *Prunus ferganensis*) × peach and found substantial marker collinearity across the different maps. Worthy of note, in these studies, occasional divergencies between maps of different species were attributed to the mapping of different duplicates of markers that have more than one copy in different regions of the *Prunus* genome. Additionally, the occasional inversion of adjacent markers was ascribed to the limited size of population samples and errors in marker order assignment [16, 17]. Practical implications of syntenic regions are highlighted in Messina *et al*. [51], who demonstrated that SSR markers can be effectively transferred across various *Prunus* species, including peach, nectarine, almond, European plum, Japanese plum, sweet cherry, and sour cherry, with 20% of the SSR markers showing successful amplification across all *Prunus* species tested.

In polyploid *Prunus* species such as *Prunus spinosa* (2n = 4x), *P. domestica* (2n = 6x), and cherry laurel (*Prunus laurocerasus*, 2n = 22x), the ‘targeted sequence by gene synteny’ approach can be particularly useful for elucidating the genome structure of complex regions associated with key traits [66]. This method gives insight into the genetic architecture of complex genomes by integrating synteny information to refine sequencing using the whole-genome sequences of diploid relatives, facilitating the identification of markers linked to genes of interest.

In conclusion, this study has presented an overview of the current state of genetic and genomic research in *Prunus*. It has provided actionable insights for *Prunus* breeding, particularly markers for QTL hotspots and syntenic regions across *Prunus* species. The findings indicate that existing research primarily focuses on fruit quality, morphology, disease resistance, and growth and development traits. By leveraging information from this study, we aim to encourage further exploration of fruit and nonfruit traits, such as ornamental and timber qualities, to broaden the benefits of the *Prunus* genus.

## Methodology

### QTL data curation in GDR

We curated *Prunus* QTLs, MTLs, and GWAS data from publications covering 25 years (from 1998 to 2023) into the GDR. Some of the information collected from each publication include contact information of corresponding author, pedigree information of population used in the study, map data (including map name, position, and linkage group), marker information, and QTL data (including the genome assembly used, traits, statistical data, colocalized markers, etc.). The algorithm used for the analysis was also recorded. Each QTL, MTL, or QTN reported in publications was given a distinct label. As a result, the same locus might have been counted multiple times if reported in different mapping populations or with varying statistics in different studies. All evaluated traits belong to at least one of the seven trait categories: morphology, quality, yield, growth and development, biochemical, stress, and fertility traits.

### *In silico* PCR to align PCR-based markers to whole genomes

Primer sequences of the polymerase chain reaction (PCR)-based (mainly SSR) markers reported in publications were aligned to whole-genome sequences using blastn-short. The four genomes used for this study were *P. persica* v2.0 [28], *P. avium* Tieton Genome v2.0 [67], *P. avium* Regina Genome v1.0 [68], and *P. dulcis* Lauranne Genome v1.0 [69]. These genomes were selected because they are commonly used as reference genomes in publications for individual *Prunus* species. The best hit was used to estimate the aligned location of the markers. The product size cutoff was 80–700 bp for SSRs and 80–2000 bp for other PCR-based markers such as expressed sequence tags, gene marker, and sequence-characterized amplified regions. PCR products that did not meet the above set criteria were excluded from the analysis.

### QTL alignment to genomes

QTLs of all *Prunus* species in this study were first aligned to the peach genome, which served as the reference genome in many *Prunus* studies. To further visualize the variations among *Prunus* species, QTLs were aligned to individual genomes listed above. When available, the genome positions of colocalized markers were used for alignment; otherwise, the positions of neighboring markers were utilized. When multiple markers were associated with a QTL, the left-most and the right-most positions were used as the start and end positions of the QTL. These data were loaded to the GDR.

### Case study: identification of QTL/MTL hotspots and syntenic regions

We downloaded *Prunus* QTL data using the MegaSearch interface in the GDR. Potential QTL/MTL hotspots in all four *Prunus* genomes were identified based on QTL/MTL proximity (in ~4 Mb) on each chromosome, except when the range of an individual QTL was wider than 4 Mb. We defined potential QTL hotspots as the existence of at least four QTLs for correlated traits within a 4-Mb region. The 4-Mb window was selected based on prior identification of QTL hotspots within 1–4 Mb in peach [11, 58]. We focused on including only QTLs and MTLs (not QTNs) in the hotspot determination since they are accompanied by readily accessible marker information to facilitate their use by breeders. For each hotspot, the following information was recorded: physical and genetic distance, study populations, maps, and colocalized markers.

Next, we searched for QTLs that are aligned to more than one crop. This was possible since we aligned peach QTLs onto the genomes of sweet cherry and almond and vice versa. We documented trait names, linkage groups, QTL labels, and regions across all four genomes (peach, almond, and two sweet cherry genomes). Additionally, colocalizing markers and PCR primers were recorded. We then analyzed the QTLs situated within the conserved syntenic regions documented in GDR. The syntenic regions were identified using MCScanX [70] with default settings—the BLAST files were generated using blastp with an expectation value cutoff of <1e-10, a maximum of five alignments, and a maximum score of 5. We specifically examined syntenic regions on Chr 4 because Chr 4 harbors QTLs from several trait categories and has many QTL hotspots in peach [11, 58, 71, 72]. Upon further examination of the syntenic blocks on Chr 4, several key genes were identified. A detailed search for orthologues and paralogues was then conducted by copying the gene coordinates from each syntenic block and pasting them on the MegaSearch interface in GDR.

## Supplementary Material

Web_Material_uhaf142

## Data Availability

All data presented in this work are available on the GDR website (ww.rosaceae.org).
